# Group B streptococci infection model shows decreased regulatory capacity of cord blood cells

**DOI:** 10.1038/s41390-021-01880-1

**Published:** 2022-02-14

**Authors:** Kriszta Molnar, Hannah Riedel, Julian Schwarz, Stefanie Dietz, Bärbel Spring, Laura Haag, Christian F. Poets, Christian Gille, Natascha Köstlin-Gille

**Affiliations:** 1 Department of Neonatology, Tübingen University Children’s Hospital, Calwerstrasse 7, 72076 Tübingen, Germany; 2 Department of Neonatology, Heidelberg University Children’s Hospital, Im Neuenheimer Feld 430, 69120 Heidelberg, Germany

## Abstract

**Background:**

Sepsis is one of the leading causes of morbidity and mortality in the neonatal period. Compared to adults, neonates are more susceptible to infections, especially to systemic infections with Group B *Streptococcus* (GBS). Furthermore, neonates show defects in terminating inflammation. The immunological causes for the increased susceptibility to infection and the prolonged inflammatory response are still incompletely understood.

**Methods:**

In the present study, we aimed to investigate the reaction of cord blood mononuclear cells (MNC) to stimulation with GBS in comparison to that of MNC from adult blood with focus on the proliferative response in an in vitro infection model with heat-inactivated GBS.

**Results:**

We demonstrate that after stimulation with GBS the proliferation of T cells from adult blood strongly decreased, while the proliferation of cord blood T cells remained unchanged. This effect could be traced back to a transformation of adult monocytes, but not cord blood monocytes, to a suppressive phenotype with increased expression of the co-inhibitory molecule programmed death ligand 1 (PD-L1).

**Conclusions:**

These results point towards an increased inflammatory capacity of neonatal MNC after stimulation with GBS. Targeting the prolonged inflammatory response of neonatal immune cells may be a strategy to prevent complications of neonatal infections.

**Impact:**

- Neonatal sepsis often leads to post-inflammatory complications.
- Causes for sustained inflammation in neonates are incompletely understood.
- We show that cord blood T cells exhibited increased proliferative capacity after stimulation with group B streptococci (GBS) in comparison to adult T cells.
- Adult monocytes but not cord blood monocytes acquired suppressive activity and expressed increased levels of PD-L1 after GBS stimulation.
- Increased proliferative capacity of neonatal T cells and decreased suppressive activity of neonatal monocytes during GBS infection may contribute to prolonged inflammation and development of post-inflammatory diseases in newborns.

## Introduction

Sepsis is one of the leading causes of morbidity and mortality in the neonatal period.^1,2^ The incidence of neonatal sepsis is about 0.05% in term neonates (≥37 weeks of gestation) but increases to up to 36% among very low birth weight infants.^3^ Although decreasing in incidence, neonatal sepsis still causes approximately 400,000 infant deaths globally.^4^

Compared to adults, neonates and especially preterm infants are much more susceptible to infections, which is reflected in an eight times higher sepsis risk.^5^ Furthermore, the termination of inflammation seems to be disturbed in neonates and preterm infants,^6^ a phenomenon named sustained inflammation.^7^ This prolonged inflammatory reaction after initial activation of neonatal immune cells may contribute to the development of post-inflammatory diseases such as bronchopulmonary dysplasia (BPD) and periventricular leukomalacia (PVL), thereby significantly influencing long-term outcome.^8–10^ Both, increased susceptibility to infection as well as prolonged inflammation, can be traced back to altered reactions of neonatal immune cells compared to those of adults.^11^ Group B *Streptococcus* (GBS) is one of the leading cause of neonatal sepsis and meningitis. Although intrapartum antibiotic prophylaxis has significantly decreased the incidence of invasive GBS infections, still about 1 in 1000 newborns suffers from GBS sepsis often associated with a poor outcome.^12,13^ Some differences between neonates and adults in the reaction of immune cells to GBS have been identified^14,15^; however, it still remains unclear why neonates and especially preterm infants are much more susceptible to invasive GBS infections than adults and why they frequently develop post-inflammatory diseases.

In the present study, we aimed to examine the response of cord blood mononuclear cells (CBMC) to stimulation with GBS in comparison to that of mononuclear cells (MNC) isolated from peripheral blood (PB; peripheral blood mononuclear cells (PBMC)). In an in vitro co-culture model, we show (1) that the proliferation of PBMC strongly decreased after stimulation with GBS while proliferation of CBMC remained unchanged and that (2) the decreased proliferation of PBMC after GBS stimulation was due to changes in monocyte and T cell function. Also, (3) monocytes from adult, but not from CB, acquired suppressive activity and a different phenotype after stimulation with GBS. (4) T cells from adult blood expressed increased levels of the activation marker CD25 but did not acquire increased suppressive activity after stimulation with GBS.

## Results

### Decreased proliferation of adult blood but not CB MNC after stimulation with GBS

**Fig. 1:**
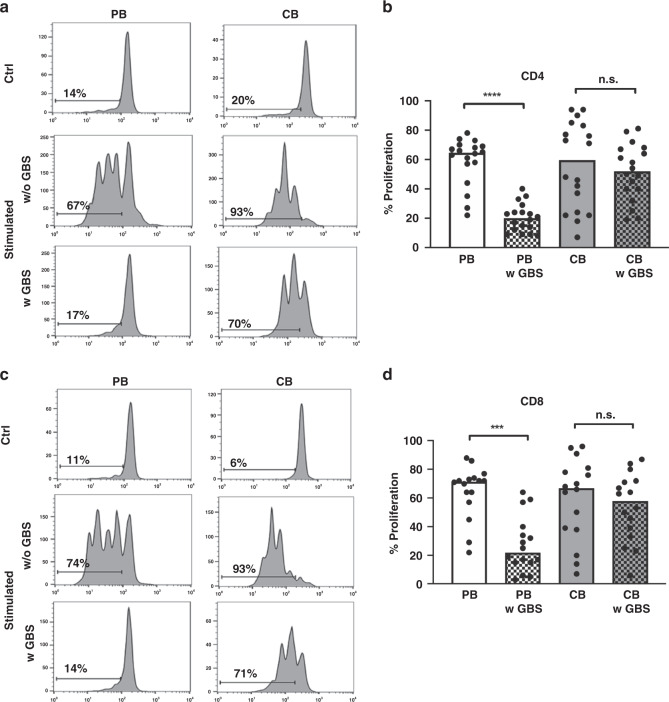
Proliferation of adult and cord blood mononuclear cells after stimulation with GBS. CBMC and PBMC were isolated, stained with CFSE and stimulated with GBS overnight. The next day, cells were stimulated with OKT3. After another 3 days, T cell proliferation was assessed by CFSE dye dilution. T cells without pre-stimulation with GBS served as control. **a**, **c** Representative histogram plots show proliferation of CD4^+^-T cells (**a**) and CD8^+^-T cells (**c**) from adult (PB) and cord blood (CB) of unstimulated cells (ctrl), stimulated cells without (w/o GBS) and with additional stimulation with GBS (w GBS). **b**, **d** Scatter plots with bars show the percentage of proliferation of CD4^+^ (**b**) and CD8^+^ (**d**) T cells in PBMC (white) and CBMC (grey) without (clean) or with (checked) stimulation with GBS. *n* = 18, ***p < 0.001, ****p < 0.0001, ns: not significant; Kruskal–Wallis test and Dunn’s multiple comparison test.

First, we asked whether stimulation with GBS would influence T cell proliferation in PBMC and/or CBMC. PBMC and CBMC were isolated from adult or CB and stimulated with GBS for 18 h. Unstimulated PBMC and CBMC served as controls. Subsequently, T cell proliferation was stimulated by addition of OKT3. We found that in adult donors, pre-stimulation with GBS led to a strong reduction in proliferation of CD4^+^ T cells (median 20.0 versus 64.5%) as well as CD8^+^ T cells (median 22.0 versus 71.5%, both *n* = 18, *p* < 0.0001) in comparison to cells not stimulated with GBS. In contrast, stimulation of CB cells with GBS did not alter CD4^+^ or CD8^+^ T cell proliferation (median 52.0 versus 59.5% for CD4^+^-T cell and 58.0 versus 67.0% for CD8^+^-T cell proliferation) (Fig. 1a–d). Stimulation with lipoteiconic acid (LTA) induced similar effects with an inhibition of CD4^+^- and CD8^+^-T cell proliferation in PBMC and a tendency towards an increased T cell proliferation in CBMC (Supplementary Fig. 2A–D).

### Decreased proliferation of adult blood MNC after GBS stimulation is due to changes in monocyte and T cell function

**Fig. 2:**
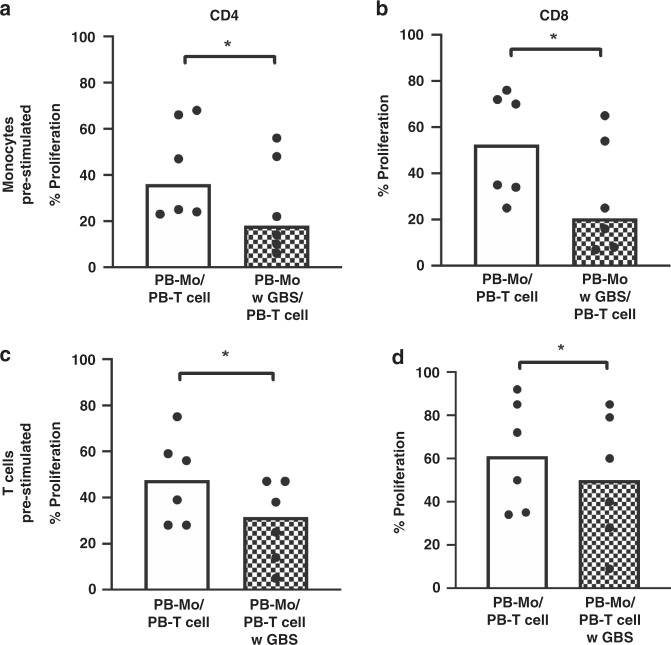
T cell proliferation in adult blood after pre-stimulation of monocytes and T cells with GBS separately. Monocytes or T cells from PBMC were enriched by MACS and stimulated with GBS overnight. The next day, freshly isolated and CFSE-stained T cells from a different adult donor were added to pre-incubated monocytes (**a**, **b**) or pre-incubated, CFSE-stained T cells were added to freshly isolated monocytes from a different adult donor in a 2:1 ratio. Non-stimulated cells served as control. Co-cultures were stimulated with OKT3, and after 72 h, T cell proliferation was assessed by flow cytometry. **a**, **b** Scatter diagrams with bars show proliferation of CD4^+^ (**a**, **c**) and CD8^+^ (**b**, **d**) T cells in co-culture with monocytes without (clean) or with (checked) pre-stimulation with GBS of monocytes (**a**, **b**) or T cells (**c**, **d**). *n* = 6, *p < 0.05, ns: not significant; Wilcoxon matched-pairs signed rank test.

As OKT3-stimulated T cell proliferation in PBMC is a result of interaction between T cells and antigen-presenting cells, we asked whether the decreased T cell proliferation in PBMC was due to changes in monocyte or T cell function. Therefore, we enriched monocytes and T cells from PBMC by magnetic activated cell sorting (MACS) and stimulated them with GBS overnight. We then added either freshly isolated T cells (to pre-stimulated monocytes) or freshly isolated monocytes (to pre-stimulated T cells) from a different donor and stimulated the co-cultures with OKT3. Here we found that both, exclusive GBS pre-stimulation of monocytes (median 19.5% (with GBS) versus 36.0% (without GBS) for CD4^+^- and 20.5% (with GBS) versus 52.5% (without GBS) for CD8^+^-T cell proliferation, Fig. 2a, b) and exclusive GBS pre-stimulation of T cells (median 31.5% (with GBS) versus 47.5% (without GBS) for CD4^+^- and 50.% (with GBS) versus 61.0% (without GBS) for CD8^+^-T cell proliferation, all *n* = 6, *p* < 0.05, Fig. 2c, d), led to a decreased CD4^+^- and CD8^+^-T cell proliferation; however, the effect after GBS pre-stimulation of monocytes was stronger. As observed for stimulation of whole CBMC, exclusive stimulation of monocytes or T cells from CB did not significantly alter T cell proliferation (Supplementary Fig. 3A–D).

### Monocytes from adult blood acquire suppressive activity after stimulation with GBS

**Fig. 3:**
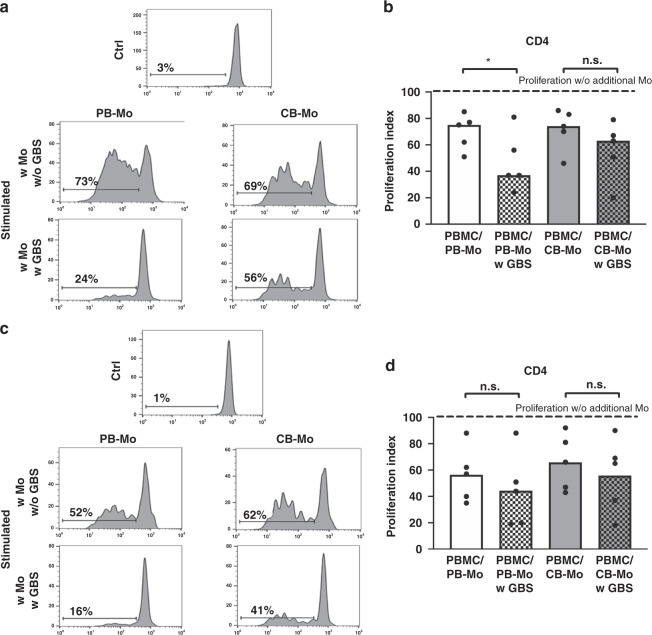
Inhibition of T cell proliferation by GBS-stimulated monocytes. Monocytes were enriched from CBMC/PBMC by MACS and stimulated with GBS overnight. Monocytes without GBS stimulation served as control. The next day, monocytes were added to freshly isolated, CFSE-stained and OKT3/IL-2-stimulated PBMC. After 4 days, T cell proliferation was assessed by CFSE dye dilution. Proliferation index was determined as the ratio of T cell proliferation with and without addition of monocytes. **a**, **c** Representative histogram plots show proliferation of CD4^+^ T cells (**a**) and CD8^+^ T cells (**c**) after addition of monocytes from PB and from CB of unstimulated cells (ctrl) and stimulated cells without (w/o GBS) and with additional stimulation with GBS (w GBS). **b**, **d** Bar graphs show the inhibitory effect of monocytes on proliferation of CD4^+^ (**b**) and CD8^+^ T cells (**c**). Dashed line shows proliferation of target PBMC without addition of monocytes. White/grey bars show T cell proliferation after addition of adult/cord blood monocytes. Clean bars show the effect of monocytes without and checked bars with pre-stimulation with GBS. *n* = 6, *p < 0.05, ns not significant; Friedman test and Dunn’s multiple comparison test.

To further investigate functional changes in monocytes after GBS stimulation, we performed inhibition assays with GBS-stimulated monocytes and freshly isolated PBMC from a different donor with the hypothesis that GBS stimulation not only decreases the co-stimulatory capacity of adult monocytes but also induces suppressive capacity. Indeed, addition of GBS-stimulated monocytes from adult blood significantly inhibited CD4^+^ proliferation from median 75% (unstimulated adult monocytes) to median 37% (GBS-stimulated adult monocytes), while CB monocytes had no suppressive effect on CD4^+^-T cell proliferation (*n* = 5, *p* < 0.05, Fig. 3a, b). CD8^+^-T cell proliferation was neither inhibited by adult nor by CB monocytes (Fig. 3c, d). Unstimulated monocytes from PB and CB had also no T cell-suppressive capacity (Supplementary Fig. 4).

### Monocytes from adult blood express increased levels of inhibitory molecules after GBS stimulation

**Fig. 4:**
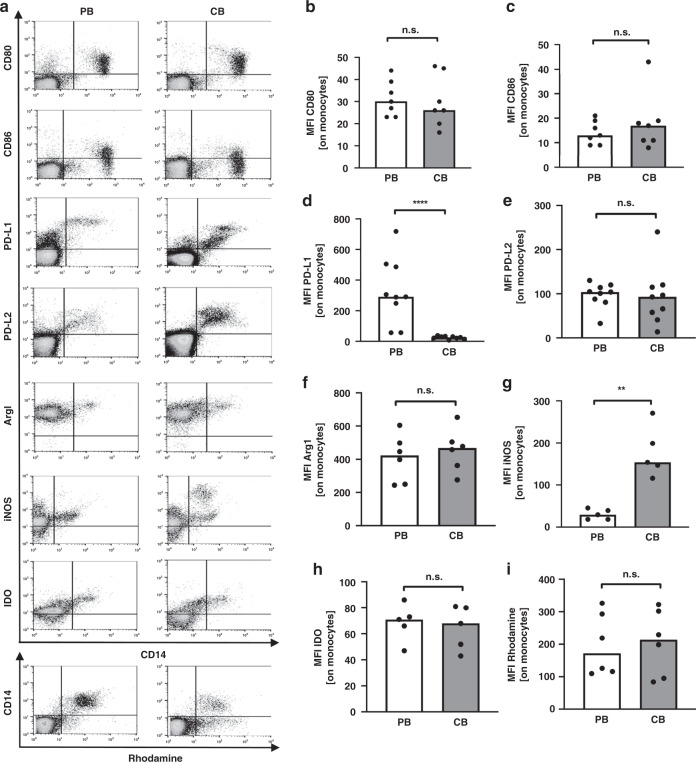
Expression of surface molecules and effector enzymes on monocytes after stimulation with GBS. CBMC and PBMC were isolated and incubated with GBS overnight. Expression of surface molecules, intracellular effector enzymes, and reactive oxygen production was determined by flow cytometry. **a** Representative density plots show the expression of CD80, CD86, PD-L1, PD-L2, ArgI, iNOS, IDO and Rhodamine (*y*-axis) on CD14^+^ adult (PB) and cord blood (CB) monocytes (*x*-axis). **b**–**i** Scatter plots with bars show the mean fluorescent intensity (MFI) for the expression of CD80 (**b**), CD86 (**c**), PD-L1 (**d**), PD-L2 (**e**) ArgI (**f**), iNOS (**g**), IDO (**h**) on and the production of ROS (**i**) by adult (white) and cord blood (grey) monocytes after stimulation of GBS. *n* = 5–9, **p < 0.01, ****p < 0.0001, ns not significant; Mann–Whitney test.

To get hints on potential mechanisms for this suppressive capacity, we analysed the expression of co-inhibitory molecules and effector enzymes associated with T cell suppression. We found that, after stimulation with GBS, adult monocytes expressed >100 times higher levels of the co-inhibitory molecule PD-L1 than CB monocytes (median mean fluorescent intensity (MFI) 291 versus 26, *n* = 9, *p* < 0.0001, Fig. 4a, d), while expression of PD-L2 (Fig. 4a, e) and co-stimulatory molecules CD80 and CD86 (Fig. 4a–c) were similar in adult and CB monocytes. Expression of arginase 1 (ArgI) and indoleamine-2,3-dioxygenase (IDO), as well as the production of reactive oxygen species (ROS) did not differ between adult and CB monocytes after stimulation with GBS (Fig. 4a, f, h, i). Interestingly, CB monocytes expressed significantly higher levels of inducible nitric oxide synthase (iNOS) than adult monocytes (median MFI 171 versus 45, Fig. 4a, g).

### T cells from adult blood express increased levels of CD25 after GBS stimulation

**Fig. 5:**
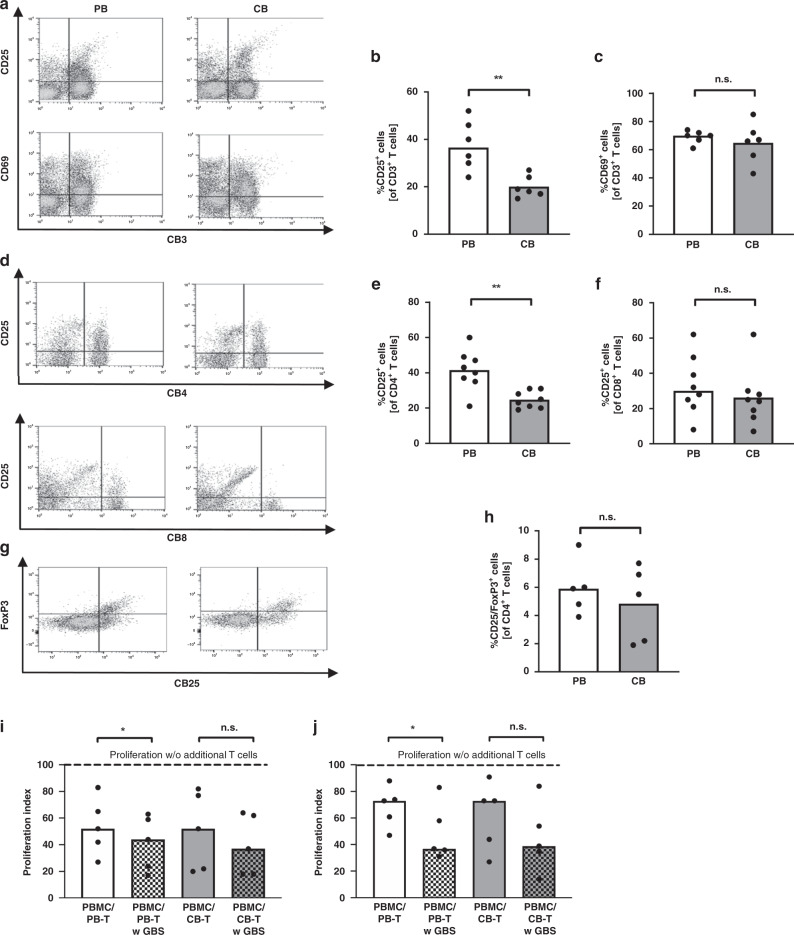
Expression of surface molecules on T cells after stimulation with GBS and inhibition of T cell proliferation by GBS-treated T cells. CBMC and PBMC were isolated and incubated with GBS overnight. Cells without GBS stimulation served as control. Expression of surface molecules (**a**–**f**) or intracellular FoxP3 (**g**, **h**) on T cells was determined by flow cytometry. Pre-incubated T cells were added to freshly isolated, CFSE-stained and OKT3/IL-2-stimulated PBMC. After 4 days, T cell proliferation was assessed by CFSE dye dilution. Proliferation index was determined as the ratio of T cell proliferation with and without addition of T cells (**i**, **j**). Representative density plots show the expression of CD25 and CD69 on CD3^+^ T cells (**a**), the expression of CD25 on CD3^+^/CD4^+^ and CD3^+^/CD8^+^ T cells (**d**), and the expression of CD25 and FoxP3 on CD3^+^/CD4^+^ T cells (**g**) from adult peripheral blood (PB) and cord blood (CB). Cells in d were pre-gated on CD3 and cells in panel **g** were pre-gated on CD3 and CD4. Scatter plots with bars show the percentage of CD3 T-cells expressing CD25 (**b**) or CD69 (**c**) percentage of CD25 on CD3^+^/CD4^+^ (**e**), CD3^+^/CD8^+^ (**f**) and percentage of CD25 and FoxP3 expresssion on CD3 and CD4 T-cells, adult (white) and cord blood (grey) T cells after stimulation of GBS. Scatter blot *n* = 6–8, **p < 0.01, ns not significant; Mann–Whitney test. Bar graphs show the inhibitory effect on proliferation of CD4^+^ (**i**) and CD8^+^ T cells (**j**). Dashed line shows the proliferation of target PBMC without addition of T cells. White/grey bars show T cell proliferation after addition of adult/cord blood T cells. Clean bars show the effect of T cells without and checked bars with pre-stimulation with GBS. *n* = 5, *p < 0.05, ns not significant; Friedman test and Dunn’s multiple comparison test.

Lastly, we aimed to analyse the phenotype of T cells after stimulation with GBS to identify a defect in adult T cell activation as a potential reason for their decreased proliferative capacity after GBS stimulation. Interestingly, we found a higher expression of the activation marker CD25 on adult T cells after stimulation with GBS compared to neonatal T cells (median 36.5% in PBMC versus 8.5% in CBMC, *n* = 6, *p* < 0.005, Fig. 5a, b), while the expression of CD69 did not differ between adult and neonatal cells (Fig. 5a, c). The increased expression of CD25 on adult cells was due to an increased CD25 expression on CD4^+^ (median 41.5% in PBMC versus 24.0% in CBMC, *n* = 8, *p* < 0.005, Fig. 5d, e) but not on CD8^+^ T cells (Fig. 5d, f). Supplementary Fig. 5 shows the percentages of CD25^+^ cells from CD3^+^ and CD3^+^/CD4^+^ cells without stimulation of GBS. Since CD25 is not only expressed on activated T cells but also on regulatory T cells (Tregs), we analysed the proportion of CD4^+^/CD25^+^/FoxP3^+^ Tregs in adult blood and CB after stimulation with GBS but found no differences (Fig. 5g, h). Interestingly, addition of CD4^+^ T cells both from adult blood and from CB to PBMC from a different donor inhibited T cell proliferation. Pre-stimulation with GBS increased the inhibitory capacity of CD4^+^ T cells; however, no differences between CD4^+^ T cells from adult and CB were observed (Fig. 5i, j).

## Discussion

Despite advances in neonatal care, sepsis in the first 28 postnatal days remains a major cause of mortality, leading to over 400,000 deaths per year. Knowledge concerning the cause(s) of this increased susceptibility to bacterial agents and on the regulation of neonatal immune functions is incomplete. Term and preterm infants with sepsis often develop long-term morbidity and neurodevelopmental impairment, such as BPD and PVL. This has been attributed to a prolonged inflammatory response following an initial activation of neonatal immune cells, called sustained inflammation. One of the most common pathogens associated with neonatal sepsis is GBS, counting for 40–50% of cases.^1,16^

First, we found that in adult cells, stimulation with GBS led to a strong reduction of T cell proliferation, while proliferation of CB T cells remained unchanged. Several groups have shown that stimulation of immune cells from human adults or from adult mice with bacterial antigens or intact bacteria led to a suppressed T cell proliferation.^17–20^ Only little data exist for neonates. Without bacterial stimulation, CB-MNC proliferate to a lesser extent than adult MNC, potentially due to a decreased co-stimulatory activity of CB monocytes.^11^ According to our present results, previous studies showed that proliferation of CBMC remained unaffected by stimulation with *Escherichia coli* but was decreased in adult PBMC.^6^ Others described even a stimulatory effect of lipopolysaccharide on T cell proliferation in neonatal blood.^21^

In neonates, several innate immune functions like neutrophil chemotaxis, respiratory burst, extracellular trap formation^22^ and monocyte proinflammatory cytokine production^23^ seem to be decreased compared to adults. However, others, like phagocytosis, are adult-like even in very preterm infants.^23^ Similar observations have been made for neonatal adaptive immunity; while Th1-responses to several pathogens and vaccines are decreased in neonates, they are able to develop adult-like Th1-responses to Bacillus Calmette–Guérin vaccination or cytomegalovirus infection.^24^ This indicates that the neonatal immune response is not generally affected and that the quality of the stimulus determines the response. Furthermore, it has been shown that there are different suppressive mechanisms present in CB, e.g. increased levels of immune-suppressive cells like myeloid-derived suppressor cells,^25^ immune-suppressive CD71^+^ erythroid cells^26^ and Tregs,^27^ as well as soluble factors like adenosine^28^ regulating the neonatal immune response. It may be speculated that the observed increased proliferative response of neonatal T cells upon GBS stimulation reflects a high inflammatory capacity regulated by suppressive mechanisms under steady state conditions, but resulting in a decreased capacity to terminate inflammation during infection, potentially leading to PVL or BPD. We further found that the decreased proliferation of adult MNC after stimulation with GBS was due to changes in monocyte as well as T cell functions. Adult monocytes acquired suppressive capacity upon stimulation with GBS while CB monocytes did not. The induction of suppressive monocytes during infection in adults has been repeatedly described.^29,30^ For neonatal cells, some studies described reduced expression of pro-inflammatory^14,31^ and increased expression of anti-inflammatory cytokines.^32^ This was traced back to high levels of soluble immune-modulatory factors like adenosine, prostaglandins and sex hormones^33,34^ perinatally. Contrarily, Roger et al. showed that the proinflammatory macrophage migration inhibitory factor MIF contributed to detrimental hyperinflammation during sepsis.^35^ Similarly, Heinemann et al. found a specific population of inflammatory monocytes in neonates that are regulated/ suppressed by S100A8/A9 alarmins.^36^ Together with our present results, it can be assumed that neonatal monocytes do not have an intrinsically decreased inflammatory capacity but that this is rather controlled by extrinsic factors.

We could not fully uncover the mechanism through which adult monocytes acquire suppressive properties upon GBS stimulation. We found a strong upregulation of PD-L1 in adult but not in neonatal monocytes. This is in line with a previous study showing increased PD-L1 expression on monocytes from septic patients and restoration of monocyte functions during sepsis after blockade of PD-L1.^37^ In vitro studies revealed that stimulation of monocytes from healthy adults with streptococcal pyrogenic exotoxin A facilitated the accumulation of Tregs through PD-L1^38^ pointing towards a regulatory role of PD-L1 expression on monocytes during infection in adults.^39^ In the present study we did not investigate any downstream pathways of PD-L1 activation, thus it can only be speculated that the increased PD-L1-expression in adult monocytes in comparison to CB monocytes contributes to the acquisition of inhibitory capacity. Expression of PD-L1 on neonatal monocytes upon bacterial stimulation has not yet been investigated. However, it seems to be relevant for the development of tolerance to allergens in the first weeks of life.^40^ Further studies are needed to investigate the role of PD-L1 for neonatal sepsis and pathogenesis of post-inflammatory diseases.

Interestingly, we found no differences in the expression of ArgI and IDO, as well as in the production of ROS between adult monocytes and CB monocytes after stimulation with GBS that could be responsible for their T cell-suppressive activity.^41^ One previous study described a lower baseline expression of IDO in CB monocytes than adult monocytes.^42^ However the overall expression of IDO in monocytes in this study was very low (<3%), while in our study all monocytes expressed IDO, indicating that IDO needs stimulation to be expressed and differences can only be detected after stimulation. Instead, we even found an increased expression of iNOS in CB monocytes in comparison to adult cells, indicating that under our conditions NO production is not primarily responsible for their suppressive activity. An increased NO production upon GBS infection/stimulation was shown to mediate disruption of blood-brain barrier and lung inflammation.^43^ After neonatal hypoxic-ischaemic brain injury, peripheral monocytes infiltrate the brain and contribute to brain injury.^44^ Monocytes also influence the onset of BPD.^45^ Thus, the increased iNOS expression in neonatal monocytes may contribute to the development of PVL and BPD and needs further investigations.

Lastly, we found that T cells and especially CD4^+^ T cells from adult blood expressed increased levels of the activation marker CD25 after stimulation with GBS in comparison to T cells from CB. CD25 is part of the receptor for interleukin-2 (IL-2R) and IL-2R knockout mice exhibit lymphoproliferation and severe autoimmunity.^46^ This is in line with our results of an increased proliferation of neonatal T cells lacking CD25 expression in comparison to adult cells. Furthermore, CD25 is expressed on Tregs, and IL-2 is required for Treg induction from conventional T cells.^46^ However, levels of Tregs in cultures after stimulation with GBS did not differ between PBMC and CBMC and there were no relevant differences in suppressive capacity between adult and CB CD4^+^ T cells after stimulation with GBS. Interestingly, stimulation with GBS induced suppressive capacity in CD4^+^ T cells isolated from CBMC and PBMC. We assume that a functional activation of Tregs by GBS stimulation is responsible for this effect. However, this functional activation seems not specific to CBMC but occurs to the same extent in PBMC. Further investigations are needed to elucidate the impact of decreased upregulation of CD25 in neonatal T cells and its contribution to immune dysfunction during neonatal sepsis.

A limitation of our study is that nothing was known about the GBS status of mothers of the infants included in this study. Thus, it cannot be excluded that a certain pre-activation of CB cells had taken place which could explain the variability in results obtained from CB. Furthermore, CB collection was anonymized and no information on sex, exact gestational age or birth mode was available—all factors that may also influence the results. Another limitation is that an immune maturation takes place over the first weeks of life that is influenced by various factors, for example breastfeeding.^47^ Since we used CB for our analyses, the influences that play a role after birth could not be considered.

In conclusion, we show that stimulation with GBS had a strong effect on CD4^+^ and CD8^+^ T cell proliferation in adult, but not in CB cells, accompanied by the acquisition of suppressive capacity and upregulation of PD-L1 in adult monocytes. Our study illustrates a novel in vitro effect by which GBS may influence neonatal immune cells. Further studies are needed to uncover the underlying mechanisms and to evaluate if these effects also play a role in vivo. Altered reactions of neonatal immune cells to GBS may not only influence the pathogenesis of neonatal sepsis, but may also contribute to the development of post-inflammatory diseases like BPD and PVL. Modulating the post-inflammatory immune regulation may be an opportunity to prevent complications of inflammation in neonates.

## Methods

### Patients

The Ethics Committee of Medical Faculty of the University of Tübingen approved this study (458/2019BO1), and CB was collected from healthy term newborns (≥37 + 0 gestational weeks, total *n* = 54) immediately after Caesarean section or vaginal delivery in a syringe and anticoagulated with heparin. Collection of CB samples was anonymized, thus, no information on sex, exact gestational age or birth mode was available. Parents gave their written informed consent. Children with intra-amniotic infection (defined according to the guideline of the German Society for Gynaecology and Obstetrics (DGGG) as maternal fever (≥38.0 °C), increased maternal inflammatory markers without any other cause (C-reactive protein >10 mg/l or elevation of white blood cell count >15,000/μl), foetal or maternal tachycardia, painful uterus and foul-smelling amniotic fluid) were excluded. PB from healthy adults was collected from adult volunteers (total *n* = 54).

### Cell isolation and culture

MNC were isolated by density gradient centrifugation according to previously described protocols.^48^ Heparinized whole blood was diluted in phosphate-buffered saline (PBS) to a total volume of 35 ml and added carefully onto 15 ml lymphocyte separation solution (Biochrom GmbH, Berlin, Germany). Cells were centrifuged and the MNC layer was collected. Cell count was determined and cells were diluted in PBS at a concentration of 2 × 10^6^ cells/ml for extracellular staining or at appropriate concentrations (see below) in RPMI supplemented with 10% foetal calf serum (FCS), 1% penicillin/streptomycin and 1% Glutamine for culturing and functional analyses.

For separation of T cells and monocytes from the PBMC and CBMC fraction, cells were labelled with human CD4^+^ T cell isolation kit or human Pan Monocyte Isolation kit (Miltenyi Biotec, Bergisch Gladbach, Germany) and separated by MACS in an autoMACS® separator according to the manufacturer’s protocol (Miltenyi Biotec).

For stimulation with GBS or LTA, cells were seeded in 500 μl of RPMI supplemented with 10% FCS, 1% penicillin/streptomycin and 1% Glutamine at a concentration of 1 × 10^6^ cells/ml in 48-well plates (Greiner Bio-one, Kremsmünster, Austria). Cells were stimulated either with GBS in a multiplicity of infection (MOI) of 1:50 or with LTA (1 µg/ml, Sigma, Munich, Germany) for 18 h. Unstimulated cells served as control.

### Bacterial culture

The culture of Group B *streptococci* was performed as previously described.^49^ Briefly, the GBS strain BSU98 was freshly grown on Columbia agar plates (Sigma, Munich, Germany) supplemented with 5% defibrinated sheep blood and spectinomycin (150 g/ml; Sigma) for 16 h. Colonies were re-suspended in PBS and bacterial number was determined spectrometrically. Bacteria were inactivated for 30 min at 70 °C. For stimulation of PBMC and CBMC, inactivated GBS were used in a MOI of 1:50.

### Flow cytometry

Antibodies used for extracellular staining of monocytes and T cells were purchased from BD Bioscience, Heidelberg, Germany (CD3-PerCp clone SK7, CD4-APC clone RPA-T4, CD8-PE clone SK1, CD14-APC clone MφP9, CD25-FITC clone 2A3, CD80-PE clone L307.4, CD86-PerCp clone 2331), Miltenyi Biotec (CD69-APC clone REA-824) and BioLegend, San Diego, CA (PD-L1-APC clone B7H-1, PD-L2-PE clone MIH1).

For intracellular staining, a total of 2 × 10^5^ extracellularly stained PBMC and CBMC were washed with FACSbuffer (PBS with 0.1% FCS, Sigma) and 0.1% sodium azide (Sigma). One hundred microlitres of Cytofix/Cytoperm (BD Pharmingen, Heidelberg, Germany) were added and cells were incubated for 10 min at 37 °C. After that, intracellular antibodies for ArgI (ArgI-PE clone 658922), IDO (IDO-PE clone 700838) (both R&D Systems, Wiesbaden-Nordenstadt, Germany) and iNOS (iNOS-Alexa Fluor 647 clone C-11; Santa Cruz, Heidelberg, Germany) were added and incubated for another 10 min at 37 °C. Cells were then washed twice with fluorescence-activated cell sorting (FACS) buffer and analysed by flow cytometry. Data acquisition was performed with a FACS Calibur flow cytometer and data were analysed via CellQuest (BD Biosciences).^48^

All antibodies were tested for their specificity by isotype control staining, when introduced in our laboratory. Isotype control antibodies used for intracellular staining of effector enzymes were Alexa Fluor 647 mouse IgG1 IC (for iNOS), PE mouse IgG2b IC (for ArgI) and PE mouse IgG1 IC (for IDO) (all from BD Bioscience). For each antibody combination, compensation with single staining was performed. For analysis of expression of co-stimulatory and co-inhibitory molecules on and expression of effector enzymes by monocytes as well as activation markers on T cells, FMO controls were run for each sample.

### ROS detection

For detection of ROS, 1 × 10^6^ PBMCs and CBMCs, pre-incubated without or with GBS in a MOI of 1:50 for 18 h, were incubated with dihydrorhodamine 123 (Sigma) in PBS for 5 min in a water bath at 37 °C. After that, cells were stimulated for 10 min with 60 ng/ml of phorbol 12-myristate 13-acetate (Sigma). Cells were washed, surface staining with anti-CD14-APC (BD Biosciences) was performed and ROS production was analysed by flow cytometry.

### T cell proliferation assay

For analysis of T cell proliferation after GBS stimulation, freshly isolated CBMC and PBMC were stained with carboxyfluorescein-succinimidyl ester (CFSE, Invitrogen, Heidelberg, Germany) according to the manufacturer’s instructions, seeded into round-bottom CD96-well plates (2 × 10^5^ cells in 100 μl media) and stimulated with GBS in a MOI of 1:50 for 18 h. Afterwards, 0.01 μg/ml OKT3 (Invitrogen/Thermo Fisher, Waltham, MA) was added. After 72 h of incubation, cells were harvested and stained with anti-CD4-APC and anti-CD8-PE. CFSE fluorescence intensity was analysed by flow cytometry. Apoptosis rates after 4 days of culture were assessed by Annexin V staining (Annexin-V-APC, BD Biosciences) and flow cytometry and revealed apoptosis rates of about 5–10% (Supplementary Fig. 1).

To analyse the impact of GBS stimulation on monocytes and T cells separately, isolated monocytes or T cells from PB and CB (each 1 × 10^5^ cells in 500 μl media in 48 well plates) were cultured alone or with GBS for 18 h (MOI 1:50). Cells were removed from the plates and cell count was assessed in a Neubauer counting chamber. In order to maintain allogeneic combinations both in CB and in adult blood, we always used different donors for monocytes and T cells. The next day, the counterpart of cells was isolated freshly from another donor.

Pre-cultured monocytes were added in a 1:2 ratio to 2 × 10^5^ freshly isolated T cells from a different adult donor stained with CFSE and stimulated with 0.01 μg/ml OKT3 in a round-bottom 96-well plate. As control, only RPMI1640 with supplements was added to the CFSE-stained cells. After 72 h, cells were harvested and stained with anti-CD4-APC and anti-CD8-PE.

Pre-cultured T cells were labelled with CFSE and stimulated with 0.01 μg/ml OKT3. Freshly isolated monocytes from a different adult donor were added as co-stimulatory cells in a 1:2 ratio to 2 × 10^5^ T cells in a round-bottom 96-well plate. After 72 h of incubation, cells were harvested and stained with anti-CD4-APC and anti-CD8-PE. CFSE fluorescence intensity was analysed by flow cytometry. Data acquisition was performed with a FACSCalibur flow cytometer and data were analysed via CellQuest (BD Biosciences).

To analyse the suppressive capacity of GBS stimulated monocytes or T cells, isolated monocytes or T cells from PB and CB (each 1 × 10^5^ cells in 500 μl media in 48-well plates) were cultured alone or with GBS for 18 h (MOI 1:50). Cells were removed from the plates and cell count was assessed in a Neubauer counting chamber. The next day, PBMC were isolated freshly from another donor. Pre-cultured monocytes or pre-cultured T cells were added in a 1:2 ratio to 2 × 10^5^ freshly isolated PBMC from a different adult donor stained with CFSE and stimulated with 0.01 μg/ml OKT3 and 100 U/ml IL-2 (R&D Systems) in a round-bottom 96-well plate. As control, only RPMI1640 with supplements was added to the CFSE-stained cells. After 72 h, cells were harvested and stained with anti-CD4-APC and anti-CD8-PE.

### Statistics

Statistical analysis was done with GraphPad Prism version 8.0. Values were tested for Gaussian distribution using D’Agostino and Pearson omnibus normality test. Differences in proliferation assays and inhibition assays with comparison of more than two groups were analysed by Friedman test and Dunn’s multiple comparison test. Differences in proliferation assays with comparison of two groups were analysed using the Wilcoxon matched-paired signed rank test. Differences in the expression of surface molecules and intracellular molecules were determined using Mann–Whitney test. A *p* value <0.05 was considered significant.

## Supplementary information

Supplementary Figures
