# Supplementary material for: Group B streptococci infection model shows decreased regulatory capacity of cord blood cells
Source: Pediatr Res. 2022 Feb 14;92(5):1407–16. doi: 10.1038/s41390-021-01880-1 (PMC9700511; doi:10.1038/s41390-021-01880-1)
Supplement: Supplementary file 1 — Supplementary Figures [file 41390_2021_1880_MOESM1_ESM.pdf]

## Supplementary Figure 1

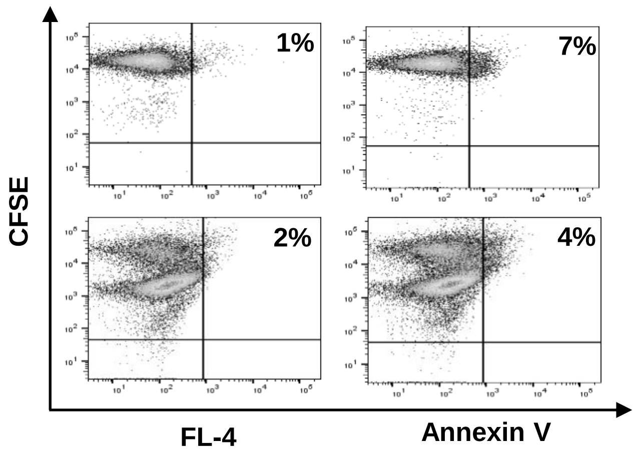


**Figure S1: Apoptosis after 96h of stimulation with GBS and OKT3**

Mononuclear cells from cord blood of term infants (CBMC) and peripheral blood of healthy adults (PBMC) were isolated, stained with CFSE and stimulated with GBS overnight. The next day, cells were stimulated with OKT3. After another three days, apoptosis was assessed by Annexin V staining. (A+C) Representative denisity plots show FL-4 (APC-channel) without antibody-staining or Annexin V-APC versus CFSE of adult blood cells (upper plots) and cord blood cells (lower plots).

## Supplementary Figure 2

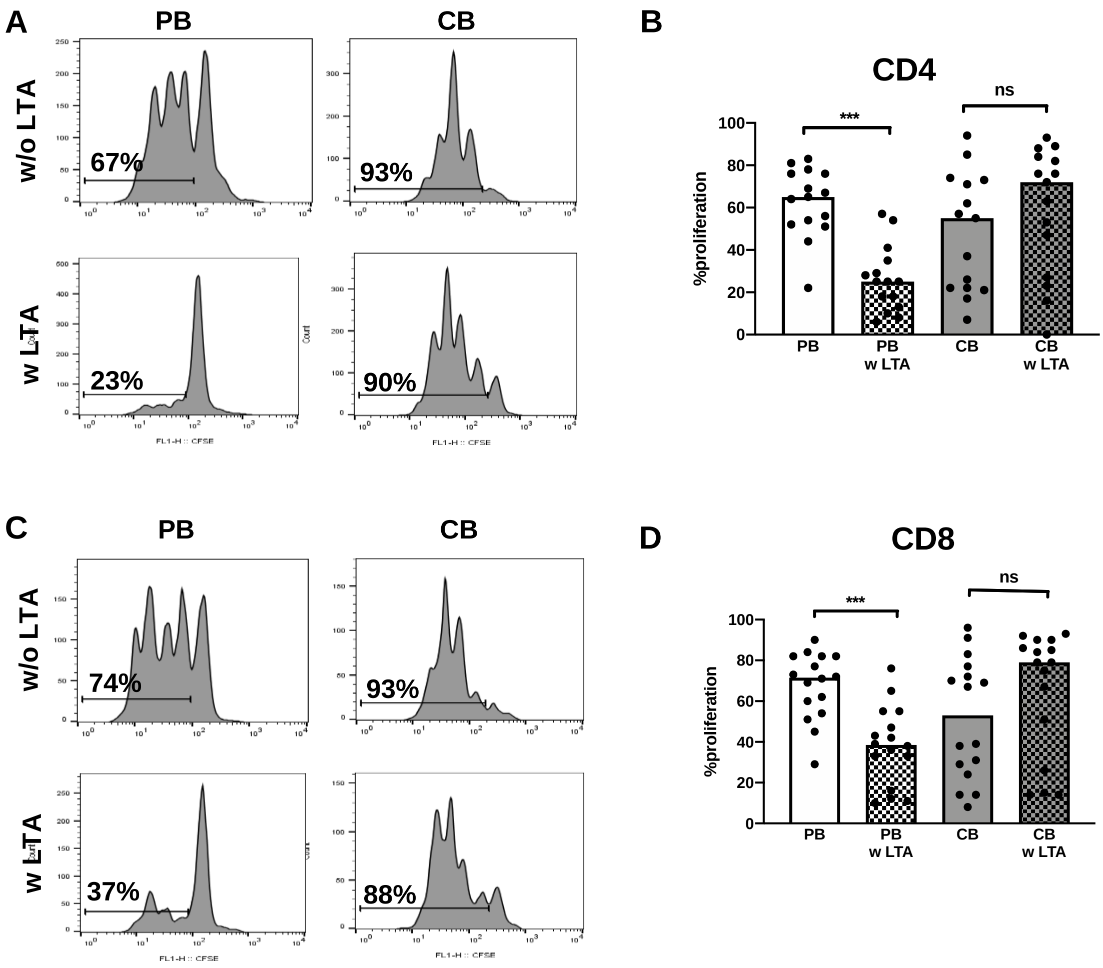


**Figure S2: Proliferation of adult and cord blood mononuclear cells after stimulation with Lipoteiconic (LTA).**

Mononuclear cells from cord blood of term infants (CBMC) and peripheral blood of healthy adults (PBMC) were isolated, stained with CFSE and stimulated with LTA overnight. The next day, cells were stimulated with OKT3. After another three days, proliferation of $CD4^{+}$ and $CD8^{+}$ T-cells was assessed by CFSE dye dilution. PBMC and CBMC without pre-stimulation with LTA served as control. (A+C) Representative histogram plots show proliferation of CD4+ (A) T-cells and CD8+ (C) T-cells from adult blood (PE, left plots) and from cord blood (CB, right plots) without (w/o LTA, same controls as shown in Figure 1) or with stimulation with LTA (w LTA). (B+D) Scatter plots with bars show the percentage of proliferation of $CD4^{+}$ (B) and $CD8^{+}$ (D) T-cells in PBMC (white bars) and in CBMC (grey bars) without (clean bars) o with (checked bars) stimulation with GBS. Bars represent pooled data from 18 independent experiments and each point represents an individual sample. *** p<0.001, **** p<0.0001, ns: not significant; Friedman test and Dunn´s multiple comparison test.

## Supplementary Figure 3

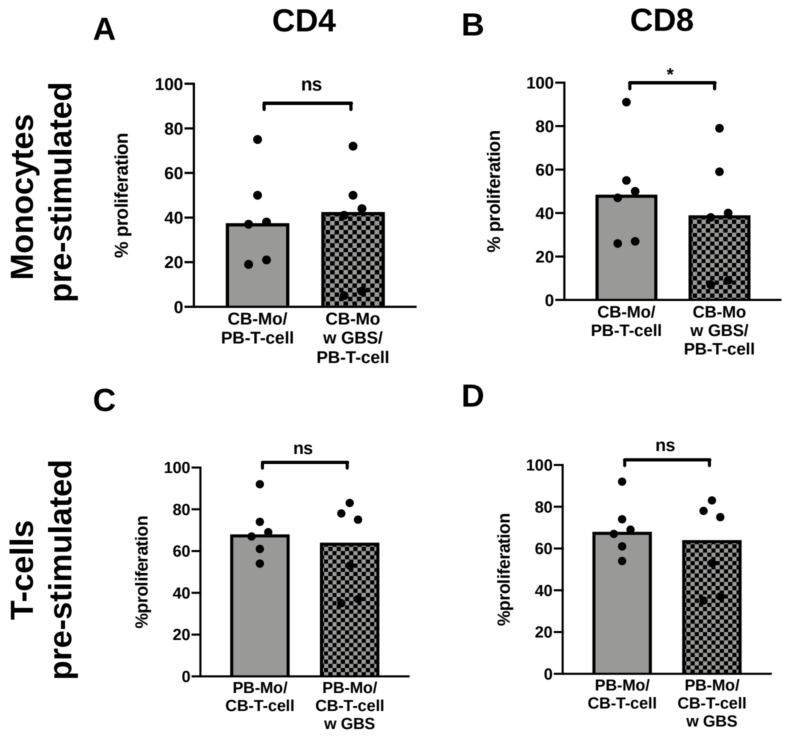


**Figure S3: T-cell proliferation in cord blood after pre-stimulation of monocytes and T-cells with GBS separately.**

Monocytes or T-cells from CBMC were enriched by MACS and stimulated with GBS overnight. The next day, freshly isolated and CFSE-stained T-cells from an adult donor were added to pre-incubated monocytes in a 2:1 ratio (A+B) or pre-incubated T-cells were CFSE-stained and added to freshly isolated monocytes from an adult donor in a 2:1 ratio. Cells without pre-stimulation with GBS served as control. Co-cultures were stimulated with OKT3 and after another 72h $CD4^+$ and $CD8^+$ T-cell proliferation was assessed by flow cytometry. (A+B) Scatter diagrams with bars show proliferation of $CD4^+$ (A) and $CD8^+$ (B) T-cells in co-culture with monocytes after pre-stimulation of monocytes without (clean bars) or with (checked bars) pre-stimulation with GBS. (C+D) Scatter diagrams with bars show proliferation of $CD4^+$ (A) and $CD8^+$ (B) T-cells in co-culture with monocytes after pre-stimulation of T-cells without (clean bars) or with (checked bars) pre-stimulation with GBS. Bars represent data from 6 independent experiments each point represents an individual sample. *$p<0.05$, ns: not significant; Wilcoxon matched-pairs signed rank test.

**Supplementary Figure 4**

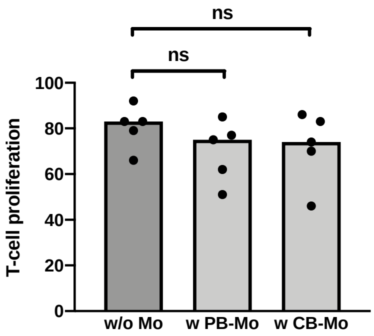


**Figure S4: Inhibiton of T-cell proliferation by pre-cultured monocytes .**

Monocytes were enriched from  cord blood of term infants (CBMC) and peripheral blood of healthy adults (PBMC) by MACS and cultured in medium overnight. The next day, monocytes were added to freshly isolated, CFSE-stained and OKT3/IL-2 stimulated PBMC. After four days, T-cell proliferation was assessed by CFSE dye dilution. Bar graph shows the T-cell proliferation without addition of monocytes (dark grey bars) and with addition of monocytes from adult blood (PB) or cord blood (CB) (light grey bars). Bars represent pooled data from 5 independent experiments and each point represents an individual sample. ns not significant; Friedman test and Dunn´s multiple comparison test.

## Supplementary Figure 5

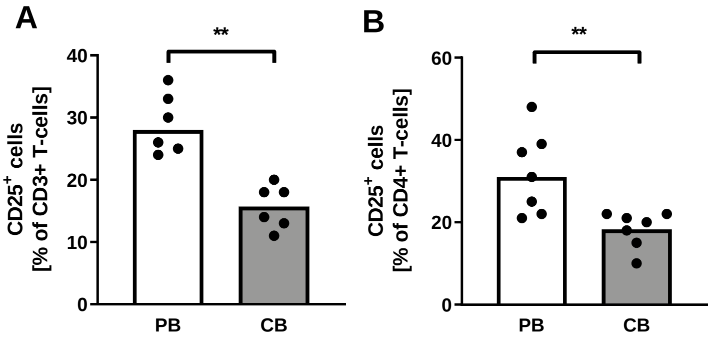


**Figure S5: Expression of surface molecules on unstimulated T-cells**

Mononuclear cells from cord blood of term infants (CBMC) and peripheral blood of healthy adults (PBMC) were isolated and incubated overnight. Expression of CD25 was determined by flow cytometry on $CD3^+$ T-cells and on $CD3^+/CD4^+$ T-cells. (A+B) Scatter plots with bars show the percentage of $CD25^+$ cells of $CD3^+$ (A) and $CD3^+/CD4^+$ cells (B). Bars represent the median of pooled data from 6-7 independent experiments and each point represents an individual sample. ** $p<0.01$, ns not significant; Mann-Whitney test.
